# Gender- and Sex-equitable Submission Guidelines in Emergency Medicine Journals Are Associated with Enhanced Publication Metrics

**DOI:** 10.5811/westjem.48527

**Published:** 2025-06-10

**Authors:** Akash Manes, Michelle D. Lall, Starr Knight, Ali S. Raja, Faisal Khosa

**Affiliations:** * University of British Columbia, Faculty of Medicine, Vancouver, British Columbia, Canada; † Emory University School of Medicine, Department of Emergency Medicine, Atlanta, Georgia; ‡ University of California, San Francisco, Department of Emergency Medicine, San Francisco, California; § Harvard Medical School and Mass General Brigham, Department of Emergency Medicine, Boston, Massachusetts

## Abstract

**Introduction:**

Gender and sex equity-promoting (GSEP) clinical research is essential to improving diversity and inclusivity in medicine. In this study we aimed to compare journal impact metrics in emergency medicine (EM) between journals that integrated gender- and sex-based considerations and those that did not.

**Methods:**

We searched the 2023 Journal Citations Report (Clarivate Analytics) for EM journals. Submission guidelines of each EM journal were examined according to the SAGER (Sex and Gender Equity in Research) guidelines and stratified as conforming or non-conforming depending on whether at least one SAGER criterion was met. Our primary outcome measure was the journal impact factor. Secondary outcome measures included other citation and influence metrics: total citations; 5-year journal impact factor; journal citation indicator; article influence score, normalized Eigenfactor score; citable items; total articles; and immediacy index.

**Results:**

Based on our classification system informed by the SAGER criteria, most journals (66%, 31/47) were classified as non-compliant. The EM journals that conformed to the sex and gender equity guidelines were rated higher than non-conforming journals across all studied journal metrics. We found that conforming journals had a significantly higher median difference (MD) than non-conforming EM journals in total citations (MD 1,586; GSEP: 3,599 vs non-GSEP: 901); 2023 2-year journal impact factor (MD 0.8; 2.3 vs 1.4); 5-year journal impact factor (MD 0.7; 2.5 vs 1.9); article influence score (MD 0.26; 0.76 vs 0.47); normalized Eigenfactor score (MD 0.79; 1.06 vs 0.26); citable items (MD 37; 103 vs 56), and total articles (MD 41; 87 vs 42). All differences were statistically significant (*P* < 0.05).

**Conclusion:**

Using criteria informed by the Sex and Gender Equity in Research guidelines, most EM journals (66%) were classified as non-conforming to these guidelines. This indicates a significant gap in the integration of gender- and sex-based considerations in EM research publication practices.

## INTRODUCTION

Gender and sex equity-promoting (GSEP) approaches aim to include all individuals, regardless of their sex or gender, while also actively addressing systemic barriers, correcting historical injustices, and ensuring fair access, participation, and outcomes for people of all gender identities and sexes. For clarity, sex is the karyotype and phenotype a person is born with, whereas gender is encompassed by gender identity, gender role, and gender expression.^1^ Gender identity is an individual’s internal sense of belonging to a particular gender; gender role is the societal expectation of how an individual should act; and gender expression encompasses the external mannerisms representing a person, which may or may not align with societal gender roles.^2^

A World Economic Forum study found that gender and sex inequities persist in economic participation and opportunity, education attainment, health and survival, and political empowerment.^2^ Recent research has highlighted disparities in academic disciplines, training programs, professional societies, editorial boards, awards, grants, and patents.^3^–^10^ In clinical research, it is essential to consider sex and gender diversity to provide equitable healthcare.^11^ However, many studies, including Cochrane reviews, disregard key patient characteristics such as sex and gender.^12^ Without inclusivity, research further exacerbates ongoing discrimination and disparities faced by those identifying as sex and gender minorities. Carefully designed study methods are necessary to ensure scientific and therapeutic discovery applicable to all sexes and genders. To promote gender and sex equity, medical journals can encourage authors to be more inclusive in their journal submission guidelines.

Previous studies have investigated gender and sex equity among submission guidelines, journal impact factor, and normalized Eigenfactor scores for radiology, ophthalmology, and obstetrics and gynecology journals; however, this research has not been conducted in the context of emergency medicine (EM) journals.^13^–^15^ In this study we analyzed the journal impact factor and Eigenfactor score as key metrics for assessing journal quality and influence. The journal impact factor is widely used for its reproducibility, while the normalized Eigenfactor score enables fair comparisons across disciplines by accounting for differences in citation trends.^16^–^21^ These metrics were analyzed for differences between EM journals that conform and those that do not conform to gender and sex equity-promoting guidelines.

Because EM is a medical discipline with diverse patient populations, it is essential to incorporate diverse perspectives to ensure gender and sex equity. This can be achieved by establishing submission guidelines that create an inclusive environment for diverse authors to share their research of diverse populations.^22^ This approach can contribute to gender and sex equity in medical research, ultimately leading to more comprehensive and applicable findings for diverse patient populations. In this cross-sectional study we investigated gender and sex equity in the submission guidelines of EM journals published in the US and internationally. Our aim was to assess whether journal impact factors and citation indices differ between conforming and non-conforming EM journals. We hypothesized that journals conforming to sex- and gender-equity guidelines would have higher metric scores than non-GSEP journals in EM.

**Population Health Research Capsule**

What do we already know about this issue?

*Many emergency medicine journals lack explicit sex and gender equity guidance. Those that include such policies may achieve stronger citation and impact metrics*.

What was the research question?

*Do emergency medicine journals with sex and gender equity guidelines have higher citation and impact metrics than those without?*

What was the major finding of the study?

*Gender- and sex-equity–promoting (GSEP) journals had a higher two-year impact factor than non-GSEP journals (median 2.3 vs 1.4; MD 0.8; 95% CI 0.2–1.8; p<0.05).)*.

How does this improve population health?

*By ensuring research reflects sex and gender diversity, evidence becomes relevant, equitable, and applicable, leading to fairer and effective population health outcomes*.

## METHODS

This cross-sectional study is reported using the Strengthening the Reporting of Observational Studies in Epidemiology (STROBE) guideline.^23^ We employed methodologies similar to those used in previously published studies investigating gender and sex equity in submission guidelines for journals in other disciplines.^13^,^14^ Lead author (AM) extracted data from the 2023 Journal Citation Report (Clarivate Analytics) in September 2024 using a standardized extraction form in Microsoft Excel (Microsoft Corporation, Redmond, WA), which conformed to the SAGER (Sex and Gender Equity in Research) guidelines, The senior author (FK) then verified the data. The study size was derived from filtered data in the ‘Emergency Medicine’ subcategory under the ‘Clinical Medicine’ category. We extracted journal name, country of publication, language of publication, publisher name, and journal metrics (impact, normalized, and source metrics) from the database. Studies were eligible for inclusion if required impact metrics, normalized metrics, and source metrics were found in the Journal Citation Report database.

We collected the following impact metrics data: total citations; 2023 journal impact factor; 5-year journal impact factor; and Immediacy Index. The journal impact factor is an approximation of the mean citation rate per citable item. The 5-year journal impact factor reflects the average citations of an article in the prior five years. In contrast, the Immediacy Index is the average citations of an article in the year of publication. We collected the following normalized metrics data: 2023 Journal Citation Indicator; Journal Citation Indicator rank; and normalized Eigenfactor and article influence scores. The Journal Citation Indicator is a category-normalized average of the citation impact for papers published in the previous three years. The Eigenfactor calculation is the number of times a publication from the prior five years was cited in 2023. In contrast, the article influence score is the Eigenfactor score multiplied by 0.01 and divided by the number of articles in the journal for normalization. For source metrics we extracted data, citable items, cited half-life, and total articles. Citable items are scholarly works that can be cited in other publications, whereas total articles are cited and non-citable. Cited half-life is the number of years that account for 50% of a journal’s total citations.

We assessed each journal’s submission guidelines for sex and gender equity using the SAGER guidelines.^24^ The SAGER guidelines comprise a comprehensive and objective tool to assess gender and sex equity in research. Among other aspects, the SAGER tool covers whether sex and gender are defined correctly, whether sex and gender differences are used appropriately, and whether sex and gender considerations for study design are explained. Although SAGER guidelines were originally developed for researchers and authors, we used it as a reference framework to assess journal submission guidelines for language that conformed to gender and sex equity. A journal was deemed GSEP if its submission guidelines satisfied any one criterion on the SAGER checklist. The SAGER guidelines are very specific; therefore, we also considered journals with editorial policies, scope statements, or guidance that aligned with at least one SAGER criterion to be GSEP. If none of the SAGER criteria were satisfied, a journal was deemed non-GSEP. Journal submission guidelines posted in languages other than English were translated using Google Translate. We excluded journals without publicly available metrics.

We used the Shapiro-Wilk test to assess normality. Since most variables were non-normally distributed, we applied quantile regression with bootstrap resampling to estimate median differences (MD) between GSEP and non-GSEP journals. This method is robust for non-normal data, provides a direct estimate of the MD, and uses bootstrapping to generate 95% confidence intervals as a measure of variability. We analyzed each covariate independently in separate quantile regression models. We assessed MD with 95% CIs for total citations, 2023 journal impact factor, 2023 Journal Citation Indicator, 5-year journal impact factor, Immediacy Index, article influence, normalized Eigenfactor score, citable items, cited half-life, and total articles. We used an alpha value of 5% to denote statistical significance. Statistical analyses were conducted in RStudio v 2024.09.0+375 (Posit PBC, Boston, MA).

## RESULTS

Of 54 journals we examined for inclusion in this study, seven were found to be ineligible. The reasons for exclusion were unavailable data on cited half-life (n = 6) and 5-year journal impact factor (n = 1). We examined submission guidelines of 47 journals and stratified them into GSEP and non-GSEP (Table 1).

Most (66%) journals did not meet one criterion of the SAGER guidelines and hence were found to be non-GSEP (31/47). Only 34% of journals were found to GSEP (16/47), as displayed in Table 1. Examples of inclusive content meeting SAGER criteria were “data should be routinely presented disaggregated by sex and gender,” “the terms sex and gender should be used correctly,” and “report how sex and/or gender were accounted for in the design of the study.”

The impact metrics data for the 47 emergency medicine journals were as follows: median total citations was 1,327 (IQR 664–3445); median 2023 journal impact factor was 1.8 (1.2–2.5); median 5-year journal impact factor was 2.0 (IQR 1.2–2.6); and median Immediacy Index was 0.5 (IQR 0.2–0.6). The normalized metrics data were as follows: median 2023 Journal Citation Indicator was 0.77 (IQR 0.47–1.07), median article influence score was 0.61 (IQR 0.31–0.78), and median normalized Eigenfactor score was 0.38 (IQR 0.18–1.02). The source metrics data were as follows: median cited items was 74 (IQR 45–120); median cited half-life 5.6 (IQR 3.8–7.0); and median total article was 73 (IQR 37–110). The median and IQR for these variables are summarized in Table 2.

Journals that conformed to gender- and sex-equity criteria outperformed non-GSEP journals across every metric: median total citations (3,599 vs 901); 2023 journal impact factor (2.3 vs 1.4); 2023 Journal Citation Indicator (1.07 vs. 0.61); 5-year outperformed non-GSEP journals across every metric: median total citations (3,599 vs 901); 2023 journal impact factor (2.50 vs 1.90), Immediacy Index (0.5 vs 0.3); article influence score (0.76 vs 0.47); normalized Eigenfactor score (1.06 vs 0.26); citable items (103 vs 56), cited half-life (5.9 vs 5.5); and total articles (87 vs 42), as summarized in Table 3. Furthermore, GSEP and non-GSEP EM journals had a significant median difference for total citations (MD: 1,586, 95% CI, 162–5,837], *P* < .05), 2023 journal impact factor (MD: 0.8, 95% CI, 0.2–1.8], *P* < .05), 5-year journal impact factor (MD: 0.7, 95% CI, 0.1–2.0, *P* < .05), article influence (MD: 0.26, CI, 0.05–0.77, *P* < .05), normalized Eigenfactor score (MD: 0.79, 95% CI (0.11, 1.32], *P* < .05), citable items (MD: 37, CI [2, 110], *P* < .05), total articles (MD: 41, 95% CI [5.0, 88.1], *P* = .11). There was no significant MD in 2023 JCI (MD: 0.41, 95% CI, −0.01, 0.88], *P* = .08), Immediacy Index (MD: 0.2, 95% CI, 0.0–0.5], *P* = .13), and cited half-life (MD: 0.4, 95% CI, −1.0, 3.7], *P* = .73) as displayed in Table 3. The reported MDs are model-based estimates derived from quantile regression with bootstrap resampling.

## DISCUSSION

Of the 47 EM journals analyzed, only 34.1% had GSEP submission guidelines. There were no journals that met all the criteria of the SAGER guidelines. The median for every assessed journal metric (total itations, 2023 journal impact factor, 2023 Journal Citation Indicator; 5-year journal impact factor, Immediacy Index, article influence, normalized Eigenfactor score, citable items, cited half-life, and total articles) was greater in GSEP journals compared to non-GSEP journals. Similarly, in ophthalmology, radiology and obstetrics and gynecology, 30%, 40% and 34% of journals, respectively, were inclusive.^13^–^15^ Furthermore, inclusive journals in these disciplines all demonstrated higher impact and influence metrics compared with their non-inclusive counterparts. 13–15

Although research into journal impact factors among GSEP and non-GSEP journals in medicine is limited, based on this work in EM and previous work in radiology, ophthalmology, and obstetrics and gynecology,^13^–^15^ these trends are expected to be generalizable across many medical disciplines. The submission guidelines of many non-GSEP journals, as defined by the SAGER criteria, across every discipline need to be re-evaluated for inclusive content. Examples of inclusive content in journal guidelines are as follows: require submitting authors to use sex/gender terminology appropriately; describe how sex/gender was considered in the study design; describe and present study populations in a sex/gender breakdown; and discuss the implications of findings and their generalizability to sex and gender minority populations.

Although GSEP journals demonstrated higher journal impact factors and normalized Eigenfactor scores, this study does not establish a causal relationship between the implementation of inclusive submission guidelines and journal impact metrics. However, our findings have significant implications for the future of medical research as they could promote a culture shift in academic medicine, encouraging journals to adopt more inclusivity in publishing practices, peer review processes, editorial policies, and institutional decision-making roles. However, it is first important to acknowledge that sex and gender inequities in academic medicine leadership continue to exist.^25^ Although these inequities cannot be resolved quickly, a significant culture shift could start if medical journals were to review their submission guidelines for inclusivity, which, in addition to ethical considerations, were motivated by the results of this study showing that GSEP journals exhibit higher journal metrics. This may significantly improve data reporting, allowing physicians to reference patient-specific data, leading to more informed and personalized healthcare decisions. Incorporating sex and gender experts into editorial boards or as reviewers is another step toward achieving this goal.

Furthermore, a broader adoption of GSEP research would also improve the overall inclusivity of academic medicine. Although women remain under-represented in senior-level academic positions even after accounting for publication-related productivity,^26^ inclusive submission guidelines may begin to address upstream inequities in recognition, authorship, and research dissemination. These improvements could support a more equitable pipeline to leadership over time, although further research is needed to confirm such long-term effects. Additionally, medicine could set a precedent for enforcing gender and sex equity in submission guidelines, hopefully followed by other fields where gender and sex equity are also critical, such as law, economics, education, and public policy research.^27^ However, since over half (63%) of GSEP EM journals were published by two publishers, Elsevier and BioMed Central, it could be suggested that publishers, in addition to journal editorial boards, influence whether submission guidelines are GSEP. Therefore, publishers and journal editorial boards are vital in creating inclusive future research practices.

A strength of this study is the comprehensive inclusion of all EM journals that report journal metrics in the Journal Citation Report database. This approach allowed for the use of normalized metrics across different journals and helped to mitigate bias.^28^ Another strength of this study is using the clear, unambiguous SAGER guidelines, which are shown to generate accurate and relevant findings to inform equitable practices.^29^

## LIMITATIONS

While there are several strengths to this study, several limitations are acknowledged. A limitation of this study is that it reviews the submission guidelines of EM journals rather than articles from each journal. Another limitation is that it only requires journals to meet one criterion of the SAGER checklist to be categorized as GSEP. Basing inclusiveness on a single, unweighted criterion makes determining the extent of a journal’s inclusiveness challenging. Furthermore, given the small sample sizes for some publishers, proportions of GSEP journals should be interpreted with caution.

Future research policies should require reputable journals to review submission guidelines in the context of gender and sex equity. Medical journals are the most reliable and utilized method to transmit, validate, and disseminate medical knowledge.^30^ Therefore, they are also primarily responsible for creating an inclusive environment conducive to continuous innovation. Without inclusive guidelines, many reports often exclude people of diverse identities, which indirectly hinders scientific discovery for people of all sexes and genders.^30^,^31^

## CONCLUSION

In this study we identified a significant lack of gender and sex inclusivity in submission guidelines for emergency medicine journals. We also found that journals promoting gender and sex equity have higher metrics than non-GSEP journals. Therefore, we recommend that the editors of EM journals review and revise submission guidelines to assist in capturing diverse research perspectives and improving journal metrics.

## Supplementary Information







## Figures and Tables

**Table 1:** Distribution of gender- and sex-equity promoting and non-GSEP promoting emergency medicine journals by publisher and country (N = 47) where GSEP journals were defined as meeting at least one sex and gender equity in research criterion.

	Total Journals (N = 47)	# of GSEP Journals (%)	# of Non-GSEP Journals (n = 31)
Total	47	16	31
Publisher			
Elsevier	10	6	4
Springer	5	0	5
Wiley	5	2	3
BioMed Central	4	4	0
Wolters Kluwer	2	1	1
Others^1^	21	3	18
Country			
USA	16	5	11
England	7	6	1
Turkey	4	0	4
Germany	4	0	4
China	3	0	3
Netherlands	2	2	0
India	2	1	1
Australia	2	0	2

1 ‘Others’ include *Pediatric Emergency Care, Western Journal of Emergency Medicine, Prehospital and Disaster Medicine, Burns & Trauma, European Journal of Trauma and Emergency Medicine, Ulusal Travma Ve Acil Cerrahi Dergisi – Turkish Journal of Trauma & Emergency Surgery, Emergencias, World Journal of Emergency Medicine, Emergency Medicine International, Archives of Academic Emergency Medicine, Open Access Emergency Medicine, Signa Vitae, Trauma Monthly, International Journal of Burns and Trauma, Emergency Medicine Journal, Trauma – England, Journal of Acute Medicine, Prehospital Emergency Care, Eurasian Journal of Emergency Medicine, Notarzt, Clinical and Experimental Emergency Medicine*.

*GSEP*, gender and sex-equity promoting; *SAGER*, sex and gender equity in research.

**Table 2:** Median and IQR for journal metrics of 47 emergency medicine journals included in a study of their adherence to gender- and equity-promoting criteria.

	Median for Included Journals (N = 47)	IQR
Total citations	1,327	664 – 3,445
2023 JIF	1.8	1.2 – 2.5
2023 JCI	0.77	0.47 – 1.07
5-year JIF	2.0	1.2 – 2.6
Immediacy Index	0.5	0.2 – 0.6
Article influence	0.61	0.31 – 0.78
Normalized EI	0.38	0.18 – 1.02
Citable items	74	45 – 120
Cited half-life	5.6	3.8 – 7.0
Total articles	73	37 – 110

*EI*, Eigenfactor*; JIF*, journal impact factor; *JCI*, Journal Citation Indicator.

**Table 3:** Median journal metrics (raw observed medians and interquartile ranges) for journals that conformed and did not conform to gender- and sex-equity promoting criteria.

	Median (IQR) for GSEP Journals (n = 16)	Median (IQR) for Non-GSEP Journals (n = 31)	Bootstrapped Median Difference (MD) [95% CI]
Total Citations	3,599 (982, 10,786)	901 (397, ,2041)	1,586 [162, 5,837]*
2023 JIF	2.3 (1.8, 3.2)	1.4 (0.8, 2.4)	0.8 [0.2, 1.8]*
2023 JCI	1.07 (0.70, 1.51)	0.61 (0.41, 0.93)	0.41 [−0.01, 0.88]
5-year JIF	2.50 (1.95, 3.50)	1.9 (0.8, 2.4)	0.7 [0.1, 2.0]*
Immediacy Index	0.5 (0.3, 0.8)	0.3 (0.1, 0.5)	0.2 [0.0, 0.5]
Article Influence	0.76 (0.56, 1.19)	0.47 (0.20, 0.66)	0.26 [0.05, 0.77]*
Normalized EI	1.06 (0.44, 1.84)	0.26 (0.09, 0.55)	0.79 [0.11, 1.32]*
Citable Items	103 (64.5, 170.5)	56 (40, 107)	37 [2, 110]*
Cited Half-life	5.9 (4.7, 9.3)	5.5 (3.8, 6.7)	0.4 [−1.0, 3.7]
Total Articles	87 (60, 145)	42 (33, 95)	41 [5.0, 88.1]*

* Statistically significant differences (*P* < .05).

*GSEP*, gender- and sex-equity promoting; *JIF*, journal impact factor; *JCI*, Journal Citation Indicator; *MD*, median difference; *EI*, Eigenfactor.
