# Supplementary material for: Gender- and Sex-equitable Submission Guidelines in Emergency Medicine Journals Are Associated with Enhanced Publication Metrics
Source: West J Emerg Med. 2025 Jun 10;27(2):465–70. doi: 10.5811/westjem.48527 (PMC13016070; doi:10.5811/westjem.48527)
Supplement: Supplementary file 1 [file wjem-27-465-s001.docx]

Appendix 1

| Publisher | Journal Name |
| --- | --- |
| BMC | Scandinavian Journal of Trauma Resuscitation & Emergency Medicine  World Journal of Emergency Surgery  BMC Emergency Medicine  International Journal of Emergency Medicine |
| Elsevier | Injury-International Journal of the Care of the Injured  Australasian Emergency Care  Resuscitation Plus  African Journal of Emergency Medicine  Resuscitation  Journal of Emergency Medicine  Journal Of Emergency Nursing  Annals of Emergency Medicine  American Journal of Emergency Medicine  Emergency Medicine Clinics of North America |
| Springer | European Journal of Trauma and Emergency Surgery  Canadian Journal of Emergency Medicine  Unfallchirurgie  Notfall & Rettungsmedizin  Current Emergency and Hospital Medicine Reports |
| Wiley | Academic Emergency Medicine  Emergency Medicine Australasia  Journal of the American College of Emergency Physicians Open  AEM Education and Training  Hong Kong Journal of Emergency Medicine |
| Wolters Kluwer Medknow | Turkish Journal of Emergency Medicine  Journal of Emergencies Trauma and Shock |
| Other | Pediatric Emergency Care  Western Journal of Emergency Medicine  Prehospital and Disaster Medicine  Burns & Trauma  European Journal of Trauma and Emergency Medicine  Ulusal Travma Ve Acil Cerrahi Dergisi – Turkish Journal of Trauma & Emergency Surgery  Emergencias  World Journal of Emergency Medicine  Emergency Medicine International  Archives of Academic Emergency Medicine  Open Access Emergency Medicine  Signa Vitae  Trauma Monthly  International Journal of Burns and Trauma  Emergency Medicine Journal  Trauma – England  Journal of Acute Medicine  Prehospital Emergency Care  Eurasian Journal of Emergency Medicine  Notarzt  Clinical and Experimental Emergency Medicine |
