# Supplementary material for: Gender- and Sex-equitable Submission Guidelines in Emergency Medicine Journals Are Associated with Enhanced Publication Metrics
Source: West J Emerg Med. 2025 Jun 10;27(2):465–70. doi: 10.5811/westjem.48527 (PMC13016070; doi:10.5811/westjem.48527)
Supplement: Supplementary file 2 [file wjem-27-465-s002.docx]

Appendix 2

| Included Journals | Pediatric Emergency Care  European Journal of Trauma and Emergency Surgery  Western Journal of Emergency Medicine  Prehospital and Disaster Medicine  Emergency Medicine Australasia  Burns & Trauma  Canadian Journal of Emergency Medicine  European Journal of Emergency Medicine  Journal of Emergency Nursing  Emergency Medicine Clinics of North America  Ulusal Travma Ve Acil Cerrahi Dergisi-Turkish Journal of Trauma & Emergency Surgery  Unfallchirurgie  Turkish Journal of Emergency Medicine  Journal of The American College of Emergency Physicians Open  Emergencias  World Journal of Emergency Medicine  Emergency Medicine International  Archives of Academic Emergency Medicine  Open Access Emergency Medicine  Notfall & Rettungsmedizin  Australasian Emergency Care  Signa Vitae  Trauma Monthly  Hong Kong Journal of Emergency Medicine  International Journal of Burns and Trauma  Trauma-England  Journal of Acute Medicine  Current Emergency and Hospital Medicine Reports  Eurasian Journal of Emergency Medicine  Notarzt  Injury-International Journal of the Care of the Injured Resuscitation  American Journal of Emergency Medicine  Annals of Emergency Medicine  Academic Emergency Medicine  Journal of Emergency Medicine  Emergency Medicine Journal  Scandinavian Journal of Trauma  Resuscitation  Prehospital Emergency Care  World Journal of Emergency Surgery  BMC Emergency Medicine  International Journal of Emergency Medicine  Aem Education X Training  Journal of Emergencies Trauma and Shock  African Journal of Emergency Medicine  Resuscitation Plus  Clinical and Experimental Emergency Medicine |
| --- | --- |
| Excluded Journals | Frontiers In Emergency Medicine  Annales Francaises De Medecine D Urgence  Emergency Care Journal  Journal of Pediatric Emergency and Intensive Care Medicine  Journal of Cardiovascular Emergencies  Journal of Endovascular Resuscitation and Trauma Management  Journal of Emergency Medicine Case Reports |
