# Supplementary material for: Gender- and Sex-equitable Submission Guidelines in Emergency Medicine Journals Are Associated with Enhanced Publication Metrics
Source: West J Emerg Med. 2025 Jun 10;27(2):465–70. doi: 10.5811/westjem.48527 (PMC13016070; doi:10.5811/westjem.48527)
Supplement: Supplementary file 3 [file wjem-27-465-s003.docx]

Appendix 3. SAGER Criteria

| Section under Author Instructions | Item Number | Checklist Item |
| --- | --- | --- |
| General | 1 | Use sex/gender terms appropriately |
| Title | 2 | Specifies to the sex/gender of participants if only one included |
| Abstract | 3a | Abstract specifies the sex/gender of participants if only one included |
| Abstract | 3b | Study population described with sex/gender breakdown |
| Introduction | 4a | If relevant, show presence or lack of sex/gender differences or similarities are cited |
| Introduction | 4b | Mention of whether sex/gender might be an important variant and if differences might be expected |
| Introduction | 4c | The demographics of the study population with regard to sex/gender (e.g., disease prevalence among male/female study participants) are outlined |
| Methods | 5a | Method of definition of sex/gender (e.g., self-report, genetic testing) |
| Methods | 5b | Description of how sex/gender was considered in the design, whether authors ensured adequate representation of male and female study participants, justification of the reasons for any exclusion of male or female participants, or explanation if not considered. Justification of other sex/gender-specific interventions of study designs (e.g., mandating contraception for women). Explicit reporting of the scientific rationale for contraception requirements and exclusions for pregnancy and lactation should be required |
| Results | 6a | Study population description with complete gender/sex breakdown for all categories considered |
| Results | 6b | Where appropriate, data presented disaggregated by sex/gender, and sex/gender differences and similarities are described |
| Results | 6c | Sex- and gender-based analyses reported regardless of outcome (in main paper if pre-specified; otherwise in appendix) |
| Results | 6d | For clinical trials, adverse event data disaggregated by sex/gender (in main paper if pre-specified; otherwise in appendix) |
| Results | 6e | Patient-reported outcome data disaggregated by sex/gender (in main paper if pre-specified; otherwise in appendix) |
| Results | 6f | For epidemiological studies, the effects of other exposures on health problems examined for all genders and analysed critically from a gender perspective |
| Results | 6g | Tables include separate rows for male sex/gender, female sex/gender and other categories if collected |
| Discussion | 7a | Potential implications of sex/gender on the study results and analyses, including the extent to which the findings can be generalized to all sexes/genders in a population |
| Discussion | 7b | If a sex/gender analysis not done, a rationale is given and implications of the lack of such analysis on the interpretation of the results are discussed |
